# Clinical and functional outcomes of the saddle prosthesis

**DOI:** 10.1007/s10195-012-0189-8

**Published:** 2012-04-17

**Authors:** D. Donati, G. D’Apote, M. Boschi, L. Cevolani, M. G. Benedetti

**Affiliations:** 1 Orthopaedic Surgery, Department of Musculoskeletal Oncology, Istituto Ortopedico Rizzoli, Via Pupilli 1, 40136 Bologna, Italy; 2 Movement Analysis Laboratory, Istituto Ortopedico Rizzoli, University of Bologna, Via di Barbiano 1/10, 40136 Bologna, Italy

**Keywords:** Saddle prosthesis, Gait analysis, Tumor surgery, Bone tumors, Resection

## Abstract

**Background:**

The implantation of a saddle prosthesis after resection of a pelvic tumor has been proposed as a simple method of reconstruction that provides good stability and reduces the surgical time, thus limits the onset of intraoperative complications. There are no studies in the literature of patients evaluated using gait analysis after being implanted with a saddle prosthesis. The present study is a retrospective case review aimed at illustrating long-term clinical and functional findings in tumor patients reconstructed with a saddle prosthesis.

**Materials and Methods:**

A series of 15 patients who recieved pelvic reconstruction with a saddle prosthesis were retrospectively reviewed in terms of clinical, radiographic, and functional evaluations. Two patients were additionally assessed by gait analysis.

**Results:**

Long-term functional follow-up was achieved in only 6 patients, and ranged from 97 to 167 months. Function was found to be rather impaired, as a mean of only 57 % of normal activity was restored. Gait analysis demonstrated that the implant had poor biomechanics, as characterized by very limited hip motion.

**Conclusions:**

Though the saddle prosthesis was proposed as advance in tumor-related pelvic surgery, the present study indicates that it yields unsatisfactory clinical and functional results due to both clinical complications and the poor biomechanics of the device. The use of a saddle prosthesis in tumor surgery did not provide satisfactory results in long-term follow-up. It is no longer implanted at our institute, and is currently considered a “salvage technique.”

**Level of evidence:**

Level IV.

## Introduction

The saddle prosthesis was developed by Nieder in 1979 at the Endo-Klinik (Hamburg, Germany) for reconstruction of severe acetabular bone defects, secondary to total replacement of an infected hip [1, 2]. Since 1984 [2], the saddle prosthesis has been also indicated as a replacement in cases with extensive resection of the periacetabular region due to bone tumors due to its design, which prevents the need for acetabular component fixation, and because it does not require a precise anatomical fit [1, 3]. This surgical option leads to a simpler method of reconstruction compared with a pelvic prosthesis or an allograft implant, and it allows hip stability and limb length to be maintained [4]. This is consistent with the reduced surgical time required, which in turn reduces intraoperative bleeding as well as the risk of infection and other perioperative complications. The basic requirements for good functional results are to adequately engrave a notch in the preserved thick and compact residual bone stock of the ilium, and to create sufficient tension between the pelvis and the femur by interposing the correct implant length. This is done to achieve properly balanced muscular tension via the iliopsoas and abductor [5]. Possible contraindications are osteoporosis, absence of residual iliac bone due to its massive involvement in the tumor [3], insufficient soft-tissue quality, as well as the absence of the psoas and the abductor muscles after tumor resection [5].

Despite the fact that the surgical technique is relatively simple, opinions on the reliability of the saddle prosthesis diverge in the current literature. Among the series present in the literature, good functional results have been reported for patients with short follow-up periods, while the results are often less favorable at longer follow-up (Table 1). A marked functional advantage is noted when implantation of a saddle prosthesis is compared to hemipelvectomy [4, 6–12]. By contrast, functional results are reported to be fair in most patients due to a limited range of motion and poor abductor muscle strength [2, 10]. On the other hand, several postsurgical complications are also described in the literature, such as deep infection [1–4, 10], wound healing disorders [3, 5, 6], dislocation [1, 2, 4–6], fractures [1, 4, 6, 10], heterotopic ossification [3, 4], nerve palsies [2, 4, 6], prosthesis cranial migration [2, 5, 10], and limb-length discrepancy [4, 6].

**Table 1:** Summary of the major series present in the literature

References	No. patients	Mean age (years)	Mean FU (months)	Major nononcological complications	Functional evaluation method	Results
De Meulemeester et al. [8]	Case report	61	60	None	Evaluation of pain and need for aids	The patient walked without pain and without the need for any support
Nieder et al. [10]	76	32–84	12–74	15 infections; 4 malpositions of the saddle; 4 dislocations; 2 fractures; 2 cases of femoral loosening	MSTS	The various parameters were reported separately and were not combined into a total score. The authors concluded that unsatisfactory results could be related to the rigid nature of the system, but that the saddle prosthesis can be used as a salvage procedure
Van der Lei et al. [11]	2 cases reported	Patient 1:68 Patient 2:43	Patient 2: 30 months	None	Evaluation of pain and need for aids	Patient 1 died 9 months after surgery. Patient 2 walked without pain and without the need for any support
Aboulafia et. al. [6]	17	59	33	3 local wound complications; 2 prosthetic dissociations; 2 dislocations; 1 secondary fracture; 1 partial sciatic nerve laceration	MSTS	9 patients were available for functional evaluation at follow-up: 7 patients showed excellent function (17–20 points in Enneking system of functional evaluation) 2 patients showed good function (14–15 points)
Windhager [12]	4	41	34	1 femoral palsy; 1 infection	MSTS	1 patient: good 2 patients: fair 1 patient: poor The mean MSTS score was 12.2 out of 30
Renard et al. [3]	15	48	36	4 deep infections; 3 fractures; 5 cases of heterotopic ossification	MSTS	Satisfactory short-term functional results. The mean MSTS score at 6 months was 50 % (no further detailed score figures are reported)
Cottias et al. [2]	17	44	42	3 infections; 2 mechanical failures; 2 sacroiliac subluxations; 4 migrations of the saddle; 3 dislocations; 3 cases of nerve damage	MSTS TESS	9 patients were available for functional evaluation at follow-up: The mean MSTS score was 57 % The mean TESS score was 58 points (range: 39–95 points)
Natarajan et al. [9]	6	37	30	1 deep infection; 1 vascular thrombosis	MSTS	3 patients: excellent 2 patients: good 1 patient: poor (No further detailed score figures are reported)
Benevenia et al. [1]	20	61	20	2 dislocations; 1 hematoma and wound necrosis	MSTS	The mean MSTS score was 55 % (No further detailed score figures are reported)
Aljassir et al. [4]	27	53	45	10 infections; 10 cases of heterotopic ossification; 6 dislocations; 6 fractures; 5 nerve palsies	MSTS TESS	14 patients were available for functional evaluation at follow-up: The mean MSTS 93 score was 50.8 % The mean MSTS 87 score was 15.3 % The mean TESS score was 64.4 %

The study described in the present paper is a retrospective case review that was performed to answer the following questions:

1. Considering the clinical, X-ray, and functional findings in tumor patients implanted with a saddle prosthesis, is this a method that is able to provide satisfactory functional results in the long term?
2. Are the poor functional results observed for the prosthesis related to its inherent biomechanics?

## Materials and methods

Fifteen patients were treated from 1995 to 2003 for pelvic resection including the acetabular area and part of the pubic rami. They were 12 males and 3 females with a mean age of 50 years (ranging from 23 to 79). Diagnosis was high-grade bone sarcoma in 6 cases, low-grade bone sarcoma in 8, and aggressive giant cell tumor in 1. In 10 patients, the saddle prosthesis was implanted as the primary reconstruction at the time of the pelvic resection, while in the other 5 it was applied following a previous failed pelvic reconstruction. In all cases, the indication for the use of a saddle prosthesis was a resection sparing part of the iliac wing as well as the glutei to achieve good saddle reconstruction stability.

During surgery, the abdominal muscles and the gluteus medius are separated from the iliac wing to gain access to the sciatic notch both internally and externally. The iliac muscle is usually sacrificed to achieve a wide tumor margin, while the psoas muscle is often saved to gain better hip stability postoperatively. Insertion of the saddle prosthesis (Waldemar Link GmbH, Hamburg, Germany) is achieved after preparing the femoral stem. The appropriate saddle length is selected before applying the prosthesis. The saddle is placed in contact with the iliac wing, engraving a notch on the medial part of the wing, while external displacement of the saddle is prevented through the use of artificial ligaments or bone graft (usually the resected femoral head). At the end of the operation, good hip stability is achieved by lengthening the operated limb.

After the drainage tubes are removed, a spica cast is applied for 25 days to improve the scar tension around the prosthesis, and then functional activity is supported for 2 months by a pelvic modular cast. Active movement and muscle rehabilitation are started after the spica cast is removed, while complete weight bearing is usually allowed 2 months after surgery. The expected functional result at the 1 year follow-up was a free walking distance without pain, supported in older patients by the use of a cane.

Usually the patients are followed up 3–4 times per year for the first 3 years after surgery, and then less strictly depending on the aggressiveness of the original pelvic tumor and any complications that occurred. During each scheduled check-up, the patient is visited and function is assessed using the Musculoskeletal Tumor Society evaluation form [13]. CT scan of the chest as well as pelvic roentgenograms are routinely taken. Sometimes, in the case of suspected local recurrence, a pelvic CT scan may also be taken.

Two patients were willing to perform gait analysis at the Movement Analysis Laboratory.

The first patient (patient 7) was affected by left periacetabular chondrosarcoma. Several muscles, such as the rectus abdominis, sartorius, rectus femoris, tensor fasciae latae, gluteus medius, and gluteus maximus were detached during surgery. The iliac muscle was resected, whereas the psoas muscle was preserved. External rotator and adductor muscles were detached and not reinserted. At the time of gait analysis (75 months postoperatively), a limb length discrepancy of about 2.5 cm was present.

The second patient (patient 4) was evaluated 60 months from the operation. The patient was affected by chondrosarcoma of the left ileopubic bone that partially extended into the acetabular region. The proximal insertion of the rectus femoris and the iliopsoas were preserved during tumor resection, whereas adductor and hip external rotator muscles were detached and not reinserted during soft tissue closure. Mild leg length discrepancy was present at gait analysis (−0.5 cm). The saddle prosthesis of this patient dislocated intrapelvically 72 months after surgery due to a trauma. At the last follow-up (167 months after surgery), the patient reported cranial migration of the prosthesis, with abundant osseous formation around the saddle component.

The instrumentation used for gait analysis consisted of a stereophotogrammetric system for motion capture (Vicon 612, Vicon Motion System Ltd., Oxford, UK) and two force plates (Kistler Instrumente AG, Winterthur, Switzerland) for recording foot–ground reaction forces. The IOR gait protocol was used to examine gait analysis variables [14]. Surface EMG was also recorded using an eight-channel system (STEP 32, DEM, Milan, Italy) that recorded at the same time as the kinematic gait analysis. The muscles explored were the bilateral erector spinae, gluteus medius, rectus femoris, biceps femoris, semitendinosus, medial gastrocnemius, and tibialis anterior.

All of the patients gave their informed consent prior being included into the study; the study was authorized by the local ethical committee and was performed in accordance with the ethical standards of the 1964 Declaration of Helsinki as revised in 2000.

## Results

The operative time ranged from 4.5 to 6 h, and the duration of pelvic reconstruction ranged from 70 to 100 min. Long-term follow-up (more than 5 years) was achieved in 6 patients (range: 97–167 months; mean: 124 months). The other patients were excluded from further evaluation due to short follow-up periods; this occurred because of early death from disease in two patients, amputation due to early infection in another two, and local recurrence in two more. Three other patients had midterm complications: one had an infection that was treated with chronic antibiotic suppression, and removal of the saddle due to continuous pain was needed in two more (Table 2). Other complications were nerve damage that healed in the first year (three patients), and persistent venous thrombosis (three patients).

**Table 2:** Demographic data for the 15 patients included in the series

Patient	Gender	Age	Implant	Side	Diagnosis	Local rec./mo	Patient follow-up (months)	Follow-up of the implant (months)	Status	Postoperative leg length discrepancy (cm)	Shortening at follow-up (cm)	Outcome
1	m	39	Primary	R	Chs	Yes/24	62	28	dec	+2	Not evaluable	Hind quarter amputation after 28 months for local recurrence, flail hip
2	m	57	Primary	L	Os	No	60	2	ned	+2	Not evaluable	Hind quarter amputation after 2 months for deep infection
3	m	40	Primary	L	Fs	No	25	25	dec	+1.5	Not evaluable	Early death from disease progression
4^a^	m	47	Primary	L	Chs	No	167	167	ned	+2	−0.5	Saddle was in site but dislocated after gait analysis was performed
5	f	56	Primary	L	Chs	No	75	75	ned	+4	Not evaluable	Deep infection occurred after 36 months; the patient refused further surgery and was treated with chronic antibiotic suppression
6	m	61	Secondary	R	Chs dediff	No	124	108	ned	0	−5	Saddle removed after 108 months for chronic pain, flail hip
7^a^	m	59	Primary	L	Chs	No	136	136	ned	0	−2.5	Saddle in site
8	m	23	Secondary	L	Os	No	126	126	ned	+1	−2.5	Saddle in site
9	m	47	Primary	R	Chs dediff	Yes/6	24	8	dec	+3	Not evaluable	Hind quarter amputation after 8 months for local recurrence
10	m	58	Primary	L	Chs	No	120	120	ned	+1.5	−3	Saddle in site
11	m	40	Secondary	R	Gct	No	101	101	ned	0	−1.5	Saddle in site
12	m	51	Primary	R	Os	No	97	97	ned	+1	−3.5	Saddle in site
13	f	25	Secondary	L	Os	No	6	6	dec	+1	Not evaluable	Early death from disease progression
14	m	79	Primary	L	Chs	No	9	3	dec	+2	Not evaluable	Hind quarter amputation after 3 months due to deep infection
15	f	68	Secondary	R	Chs	No	78	38	ned	+1.5	−2.5	Saddle removed after 38 months for chronic pain, flail hip
Range		23–79					6–167	2–167		0–4	−0.5 to −5	
Mean		50					81	69		+1.5	−2.6	
SD		15					50	56		1.1	1.3	

*Os* osteosarcoma, *Gct* giant cell tumor, *Chs* chondrosarcoma, *Chs dediff* chondrosarcoma dedifferentiated, *Fs* fibrosarcoma, *dec* dead, *ned* still alive

^a^Patients 7 and 4 were evaluated using gait analysis 75 and 60 months after the index operation

**Fig. 1a–b:**
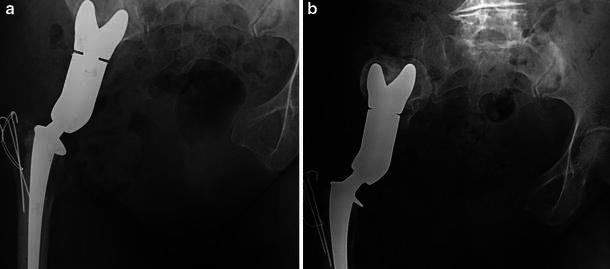
X-ray films of patient P.L., 68 y.o. at the time of operation.**a** Postoperative AP view of the pelvis; the saddle is seated through a notch in the iliac wing.**b** Same view 38 months afterwards. The patient reported suffering from pain during weight bearing for the previous 2 years. Note the proximal migration of the saddle in relation to the wearing of the iliac wing

At the radiographic evaluation, marked bone reaction around the saddle was evident after the first year of free walking in all patients. In two aged patients who had a saddle inserted as a secondary procedure, wearing of the iliac bone led to increasing pain and the need to remove the saddle, resulting in flail hip (Fig. 1a–b).

Functional evaluation (Table 3) showed relatively low scores for all of the MSTS items (mean score 17.2, 57 % of normal function). Looking at specific criteria, slight pain was always present in the older patients, although it was not disabling. Function was usually compromised, ranging from the presence of restrictions on recreational activities to major disability leading to restrictions on occupational activities, depending on the age of the patient. None of the patients wore a brace over the long term, but most of the patients preferred to use a cane for outdoor walking to avoid suffering from a marked limp or increasing pain.

**Table 3:** Functional evaluation according to the MSTS system of the 6 patients who achieved long-term follow-up (range: 97–67; mean: 124 months)

Patient	Age at diagnosis	Follow-up (months)	Pain	Function	Emotional acceptance	Support	Gait	Walking ability	Total score	%
4	47	167	4	2	3	2	2	4	17	56.6
7	59	136	4	3	3	2	2	3	17	56.6
8	23	126	5	3	2	2	2	3	17	56.6
10	58	120	4	3	3	2	2	4	18	60
11	40	101	5	3	3	3	2	4	20	66.6
12	51	97	3	2	2	2	2	3	14	46.6
Range	23–59	97–167	3–5	2–3	2–3	2–3	–	3–4	14–20	46.6–66.6
Mean	46.3	125	4.2	2.7	2.7	2.2	2	3.5	17.2	57.2
SD	13.4	25.6	0.8	0.5	0.5	0.4	0	0.5	1.9	6.5

Patients 4 and 7 were evaluated using gait analysis

The two patients evaluated using gait analysis demonstrated slow gait, asymmetric stride length, increased double support and arrhythmic stance duration, and a reduced stance on the operated side (Table 4). Hip sagittal motion was reduced in both patients.

**Table 4:** Time and distance parameters in the two patients evaluated using gait analysis

	Patient A		Patient B	
	Operated side	Healthy side	Operated side	Healthy side
Stance (%)	59.06 ± 5.29	73.7 ± 0.2	52.94 ± 1.39	71.52 ± 1.77
Stride length (m)	0.85 ± 0.09	0.82 ± 0.1	1.05 ± 0.04	1 ± 0.01
Stride time (s)	1.14 ± 0.03	1.16 ± 0.02	1.37 ± 0.06	1.35 ± 0.1
Double support (s)	33.9 ± 5.48		25.25 ± 1.41	
Cadence (stride/min)	52.08 ± 1.35		44.18 ± 2.46	
Walking speed (cm/s)	72.72 ± 7.11		75.93 ± 5.95	

Patient 7 showed a large trunk inclination (Trendelenburg gait) associated with a marked downward pelvic lean toward the operated side during the stance phase. Consistent with this pattern, a reduction in the adduction moment at the hip was found to be associated with continuous EMG activity of the erector spinae contralateral to the operated side, while the ipsilateral erectors were relatively inactive. However, EMG showed “in-phase” activity of the gluteus medius during stance. Hip motion limitation in the sagittal plane was further compensated by an increased pelvic tilt.

Patient 4 showed increased anterior trunk leaning associated with a large pelvic tilt, which increased posteriorly at initial and terminal stance to assist with foot contact. During swing, increased pelvic elevation associated with anterior rotation on the operated side was evident. EMG evidenced intense activation of both erector spinae during the swing phase. “Out-of-phase” activity of the hamstrings was present at terminal stance, which occurred in order to assist with the push-off phase.

## Discussion

In our unit, we have experienced different types of pelvic resections and reconstructions. Over the last three decades, we have tried to assess the best method of reconstruction, from iliofemoral coaptation to complex allograft–prosthetic composite reconstruction [15, 16]. However, more complex reconstructions lead to more complications after surgery, such as nerve and vessel damage, wound slough and deep infection, and mechanical or biological failure of the reconstruction [7, 17–22]. Hence, a simpler method of reconstruction—the saddle prosthesis—has been readily accepted. Surgical application of the saddle has mainly been indicated in older patients, or in difficult resections where the long operative time can increase intraoperative complications such as hemorrhage or cardiac problems.

Limitations of this study relate to the fact that it is a retrospective review with no comparison to a control group. Patients that need a pelvic resection are relatively rare, and there are many variables involved, including age, type of tumor, extent of bone and soft tissue resection, and application of adjuvant therapies (chemotherapy and radiotherapy). On the other hand, we were only able to evaluate two patients using gait analysis because of difficulties in getting other patients to participate. However, this is the first study in the literature that has reported gait analysis evaluation of patients with a saddle prosthesis.

The implantation of the saddle prosthesis is a relatively simple task, as demonstrated by the operative time, but it does lead to a significant number of postoperative complications. The most threatening was postoperative deep infection, which resulted in immediate hindquarter amputation in two cases. This is probably due to the dead space around the saddle, which can lead to hematoma even when postoperative drainage tubes are present. The rates of other complications, such as nerve palsy and venous thrombosis, could be considered in their normal ranges for this type of surgery. When we looked at the relation of the iliac bone to the saddle component, we observed progressive erosion leading to a shortening of the leg over time. The implantation of a saddle as a secondary reconstruction during old age can result in more aggressive resorption of the iliac wing. In both of the cases that required saddle retrieval, the strong and fast onset of this complication was associated with severe functional pain.

Functional evaluation of the patients who achieved long-term follow-up (average 124 months) generally yielded fair results (mean 57 % of normal). MSTS assessment indicated mild functional pain in the majority of the patients along with limited function, mainly due to the restricted motion of the hip joint. During walking, patients reported that they were rather uncomfortable, and preferred to use a cane most of the time for outdoor activities. The use of cane clearly decreased the MSTS score, including the category of emotional acceptance. This picture is consistent with some of the previous reports in the literature [7, 17], but the explanation was not very clear.

After a few reports on a small number of patients [8, 11], the use of the saddle prosthesis was popularized by the work of Nieder et al. [10]. Their series included 76 patients, and the different parameters were reported separately and not combined into a total score. They concluded that the unsatisfactory results reported could relate to the rigid nature of the system. Aboulafia et al. [6] reported excellent results in 7 patients among the 9 evaluated, and Natarajan et al. [9] noted that excellent results were obtained in 3 patients out of 5, but the mean follow-up in both studies was only 30 months. More recent studies [1, 2, 4] have reported mean MSTS scores of 57, 55, and 50.8 %, respectively, similar to the score (57 %) we observed at long follow-up.

Objective functional assessment with gait analysis allowed us to conduct a deeper biomechanical analysis of the two evaluated patients. Gait was found to be slow, asymmetric, and arrhythmic (limp gait). Individual kinesiologic adaptations were present due to the very limited hip kinematics resulting from the muscles injuries incurred during surgery and the lower limb length discrepancy. The operated limb showed limited stability under loading and problems with advancement.

Patient 7 presented a Trendelenburg gait pattern. However, even if this can be partially explained by the shorter operated limb of this patient, the presence of regular gluteus medius activity in stance can be related to a lever arm problem [10] due to eccentric displacement of the hip rotation center, making it more elevated than normal (causing wear to the iliac wing).

**Fig. 2:**
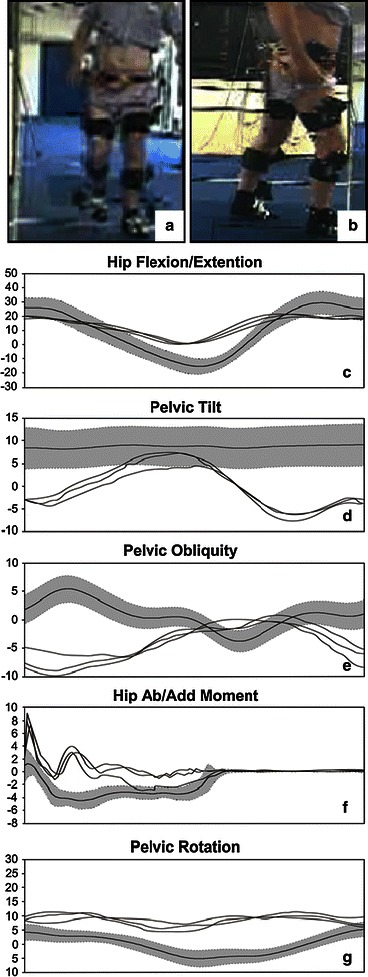
Data of patient 7 are reported (**a**–**b**), respectively hip flexion/extension (**c**), pelvic tilt (**d**), pelvic obliquity (**e**), hip adduction/abduction external moment (**f**), and pelvic rotation (**g**). The *grey band* represent the control data (mean ± 1SD, IOR Laboratory data), the *black lines* are relative to three gait trials of the left operated side

**Fig. 3:**
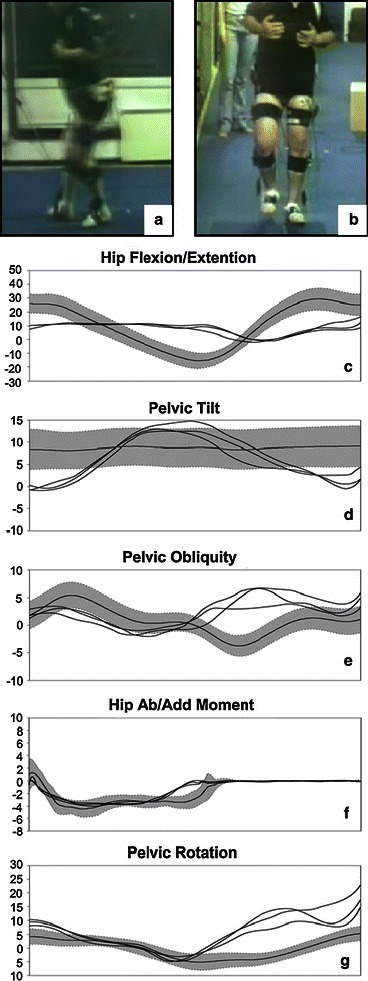
Data of patient 4 are reported (**a**–**b**), respectively hip flexion/extension (**c**), pelvic tilt (**d**), pelvic obliquity (**e**), hip adduction/abduction external moment (**f**), and pelvic rotation (**g**). The *grey band* represent the control data (mean ± 1SD, IOR Laboratory data), the *black lines* are relative to three gait trials of the left operated side

Patient 4 showed a “pelvic hike” gait pattern, consistent with a problem managing the advancement of the operated limb, even though the hip flexors were untouched during surgery. This was also confirmed by the “out-of-phase” activity of the hamstrings at the end of the stance phase, which was aimed at assisting foot detachment from the ground (Figs. 2, 3).

In conclusion, saddle prosthesis—although a simple method of pelvic reconstruction that reduces surgical time—demonstrated poor clinical and functional results in the cases we reviewed. Even if leg length can be normalized, signs of hip instability can be present due to the relationship of the saddle to the iliac wing. This method of pelvic reconstruction has, however, been useful to us as a “salvage procedure” after local recurrence or deep infection of a bulk allograft.

Gait analysis provided evidence of very limited motion and relevant compensatory gait patterns in the operated hip. This is due to the poor biomechanics of the implant, which is unable to work according to its design whatever the context of the residual musculoskeletal system.
